# AMPK Activity Contributes to G2 Arrest and DNA Damage Decrease via p53/p21 Pathways in Oxidatively Damaged Mouse Zygotes

**DOI:** 10.3389/fcell.2020.539485

**Published:** 2020-09-08

**Authors:** Pei He, Zhiling Li, Feng Xu, Gaizhen Ru, Yue Huang, En Lin, Sanfeng Peng

**Affiliations:** ^1^Department of Reproductive Center, The First Affiliated Hospital of Shantou University Medical College, Shantou, China; ^2^Guangdong Key Laboratory of Medical Molecular Imaging, The First Affiliated Hospital of Shantou University Medical College, Shantou, China; ^3^Laboratory of Molecular Cardiology, The First Affiliated Hospital of Shantou University Medical College, Shantou, China; ^4^Department of Respiratory Medicine, The First Affiliated Hospital of Shantou University Medical College, Shantou, China

**Keywords:** DNA damage, G2 arrest, oxidative stress, AMPK, p53, p21, *in vitro* fertilized embryos

## Abstract

In zygotes, the capacity of G2/M checkpoint and DNA repair mechanisms to respond to DNA damage varies depending on different external stressors. In our previous studies, we found that mild oxidative stress induced a G2/M phase delay in mouse zygotes fertilized *in vitro*, due to the activation of the spindle assembly checkpoint. However, it is unclear whether the G2/M phase delay involves G2 arrest, triggered by activation of the G2/M checkpoint, and whether AMPK, a highly conserved cellular energy sensor, is involved in G2 arrest and DNA damage repair in mouse zygotes. Here, we found that mouse zygotes treated with 0.03 mM H_2_O_2_ at 7 h post-insemination (G1 phase), went into G2 arrest in the first cleavage. Furthermore, phosphorylated H2AX, a specific DNA damage and repair marker, can be detected since the early S phase. We also observed that oxidative stress induced phosphorylation and activation of AMPK. Oxidative stress-activated AMPK first localized in the cytoplasm of the mouse zygotes in the late G1 phase and then translocated to the nucleus from the early S phase. Overall, most of the activated AMPK accumulated in the nuclei of mouse zygotes arrested in the G2 phase. Inhibition of AMPK activity with Compound C and SBI-0206965 abolished oxidative stress-induced G2 arrest, increased the activity of CDK1, and decreased the induction of cell cycle regulatory proteins p53 and p21. Moreover, bypassing G2 arrest after AMPK inhibition aggravated oxidative stress-induced DNA damage at M phase, increased the apoptotic rate of blastocysts, and reduced the formation rate of 4-cell embryos and blastocysts. Our results suggest the G2/M checkpoint and DNA repair mechanisms are operative in coping with mild oxidative stress-induced DNA damage. Further, AMPK activation plays a vital role in the regulation of the oxidative stress-induced G2 arrest through the inhibition of CDK1 activity via p53/p21 pathways, thereby facilitating the repair of DNA damage and the development and survival of oxidative stress-damaged embryos. Our study provides insights into the molecular mechanisms underlying oxidative-stress induced embryonic developmental arrest, which is crucial for the development of novel strategies to ensure viable embryo generation.

## Introduction

Eukaryotic cell cycle progression is controlled by a series of checkpoints, which ensures that cells only progress to the next stage if they are in a suitable condition, with an emphasis on DNA integrity (Canaud and Bonventre, 2015). Cells commonly trigger cell cycle checkpoints in response to DNA damage, which results in the cell cycle being halted and DNA repair pathways activated (Bartek and Lukas, 2007). If checkpoint mechanisms do not function correctly when DNA damage occurs, the cell cycle does not stop and cells are not able to effectively repair their DNA, leading to the accumulation of DNA damage and cell death. There are four major checkpoints in eukaryotic cells: G1/S, intra-S, G2/M, and intra-M, also known as the spindle assembly checkpoint.

The G2/M checkpoint prevents cells from entering mitosis unless DNA replication/repair is complete and the cell is of an acceptable size. The activity of the cyclin-dependent kinase 1 (CDK1)/Cyclin B1 complex is critical for cells entering mitosis, which is also the target of pathways that mediate G2 arrest controlled by the G2/M checkpoint (Shaltiel et al., 2015). The activity of the complex is tightly regulated by various mechanisms, including p21 activity, which is a cyclin-dependent kinase inhibitor (CKIs) (Gire and Dulić, 2015). p21 can block cell cycle progression and keep cells in either G1 (Sherr and Roberts, 1999) or G2 phase (Bunz et al., 1998; Charrier-Savournin et al., 2004). The tumor suppressor p53 plays a crucial role in DNA damage response, which upregulates the expression of several genes implicated in both G1/S and G2/M transitions (Levine, 1997; Bunz et al., 1998), including p21. Moreover, p21 plays an important role in decreasing DNA damage via inhibition of cell proliferation (Viale et al., 2009; Xu et al., 2015). Different studies have demonstrated that p21 can mediate cell cycle arrest in G2 in preimplantation embryos (Adiga et al., 2007) and fertilized eggs (Viale et al., 2009; Wu et al., 2011).

The phosphorylation of H2AX on serine residue 139 (Ser 139) (γH2AX) is a marker of the presence of DNA double-strand breaks (DSBs); this phosphorylation is mediated by the ataxia-telangiectasia mutated kinase (van Attikum and Gasser, 2005). When DSBs occur, γH2AX concentrates on DSB sites and interacts with several repair proteins that have BRCA1 COOH terminal domains (Fernandez-Capetillo et al., 2004), which play a key role in DNA damage repair. For this reason, γH2AX is widely used as a marker of DSB damage and repair.

In mouse zygotes, G2/M checkpoints and DNA repair mechanism functions are thought to be absent or compromised (Shimura et al., 2002; Yukawa et al., 2007; Toyoshima, 2009). Studies on zygotes fertilized with X-irradiated sperm have demonstrated that they do not have the traditional G2/M checkpoints (Shimura et al., 2002; Toyoshima, 2009). Yukawa et al. (2007) reported the presence of a G2/M checkpoint, but they discovered that its functionality was limited to zygotes treated with γ-irradiation, and this DNA repair mechanism appeared to be incomplete, as γH2AX was not detected in γ-irradiated zygotes. Gawecka et al. (2013) found that mouse zygotes fertilized with sperm containing severe DNA damage, induced by divalent cations treatment, triggered a G2 delay and γH2AX foci formation. In our previous study we showed that a G2/M checkpoint and DNA repair mechanism might be effective in mouse zygotes fertilized with oxygen-stressed sperm (Wang et al., 2013). These data suggest that the capacity of mouse zygotes to repair DNA damage varies in response to different external stressors.

The adenosine monophosphate (AMP) – activated kinase (AMPK), a serine/threonine kinase, is the principal energy sensor in the cell, playing a critical role in maintaining energy homeostasis (Hardie, 2007). The heterotrimeric AMPK contains a catalytic α subunit, and two regulatory subunits, β and γ (Novikova et al., 2015). This kinase is biologically inactive unless it is phosphorylated at a specific threonine residue (Thr172) in the α subunit (Sanz, 2008; Novikova et al., 2015) by upstream kinases, as well as allostery caused by AMP binding. Upon its activation, AMPK confers protection against physiological and pathological stress by upregulating metabolism to increase cellular energy and by suppressing various cellular processes to save energy (Hardie et al., 2012; Herzig and Shaw, 2017; Pei et al., 2018). In addition, AMPK was shown to regulate the cell cycle and facilitate cell survival in response to DNA damage (Sanli et al., 2010, 2014; Xu et al., 2015). However, the role of AMPK in zygotes arrested in G2 due to oxidative stress remains to be elucidated.

One feature of *in vitro* fertilization (IVF)-derived embryos is the high frequency of early developmental failure, due to differences between *in vitro* culture conditions and the *in vivo* environment. Any subtle differences in culture conditions, including culture medium (pH and contained substances), light, temperature, and gas phase, can lead to increased concentrations of reactive oxygen species (ROS) in embryonic cells (Liochev, 2013; Cui et al., 2015; Latham, 2015). The excess of ROS plays a pivotal role in DNA damage, embryo arrest, and cell death of preimplantation embryos (Takahashi, 2012). In the clinic, IVF-derived embryos subjected to excessive ROS exposure can appear normal by day 3 but have a low blastocysts formation rate (Ventura-Junca et al., 2015). Our previous studies used different doses of hydrogen peroxide (H_2_O_2_) to treat mouse zygotes at 7 h post-insemination (hpi), and found that 0.03 mM H_2_O_2_ was the minimum concentration required to generate elevated ROS levels and cause oxidative damage, which reduced the rate of blastocyst formation but did not affect the formation rate of 2-, 4-, and 8-cell embryos (Qian et al., 2016; Wu et al., 2017). We concluded that this was the condition most similarly to the physiological oxidative damage observed in the clinical practice. Our previous results also showed that 0.03 mM H_2_O_2_ induced DNA damage and caused a G2/M delay in mouse zygotes. Moreover, the delay occurred during the M phase due to the activation of the spindle assembly checkpoint (Zhang et al., 2016; Wu et al., 2017). However, these results did not confirm the existence of G2 arrest in zygotes subjected to oxidative stress, which is the typical mark of activation of the G2/M checkpoint.

In this study, we investigated the capacity of the G2/M checkpoint and DNA repair mechanisms in mouse zygotes subjected to mild oxidative stress, and the potential role of AMPK in cell cycle regulation and DNA damage.

## Materials and Methods

### Animals and Ethics

Kun-Ming mice (3–6 weeks old) were purchased from Beijing Vital River Laboratory Animal Technology. Animals were handled under the International Guiding Principles for Biomedical Research Involving Animals (2012 version), issued by the Council for the International Organizations of Medical Sciences. All experimental protocols were approved by the Laboratory Animal Ethics Committee of our institution (SUMC2019-381). This study was authorized by the Institutional Animal Care and Use Committee of Shantou University Medical College.

### Sperm and Oocyte Collection, *in vitro* Fertilization, and Embryo Culture

As described previously (Qian et al., 2016; Zhang et al., 2016), sperm was collected from the epididymis and vas deferens of male mice and incubated in capacitation medium [HTF medium (Sage Science, United States) containing 1.5% bovine serum albumin (BSA)] at 37°C in a 5% CO_2_ incubator for 1 h. To induce ovulation, female mice were injected with 10 IU pregnant mare serum gonadotropin, followed by 10 IU human chorionic gonadotropin (HCG) 48 h later. The female mice were then euthanized 13∼15 h after HCG administration, to obtain the cumulus oocytes from the oviducts. Cumulus oocytes were collected in microdrops of fertilization medium (HTF medium containing 0.4% BSA) under mineral oil (Sigma, M8410), before 10 μL capacitated spermatozoon was added to each drop. Samples were incubated at 37°C for 6 h in a 5% CO_2_ incubator, to allow for fertilization to occur. Zygotes were then transferred into embryo culture medium (HTF medium containing 0.4% BSA and 10% FBS) for further culture in a 37°C, 5% CO_2_ incubator.

### Mouse Zygote Model for Oxidative Damage

The oxidative stress-damaged mouse zygote model was established as described previously (Qian et al., 2016; Zhang et al., 2016). Zygotes were incubated in embryo culture medium containing 0.03 mM H_2_O_2_ at 7 hpi for 30 min at 37°C, then washed and further cultured with fresh embryo culture medium for subsequent experiments.

### Onset and Endpoint of S Phase and the Endpoint of M Phase of Mouse Zygotes

The onset and endpoint of S phase was determined using the BrdU incorporation experiment described previously (Zhang et al., 2016). The experiment was conducted over two time periods, 8 to 11 hpi and 16 to 19 hpi. During those times, BrdU incorporation was measured every other hour by incubating the zygotes in 1 mM BrdU-containing embryo culture medium for 30 min. Then, zygotes were fixed in 2.5% paraformaldehyde for 15 min, placed on polylysine slides, incubated in 1 mM HCl for 30 min, and then washed with 0.1 mM borate buffer solution for 20 min. Cells were permeabilized by washing three times with PBS containing 10% FBS and 0.2% Triton X-100, then blocked in the same solution for 30 min at 37°C. Zygotes were subsequently incubated with 6 μg/mL anti-BrdU antibody (Sigma, B8434) for 1 h at 37°C, rinsed thrice with PBS, and treated with secondary Cy3-conjugated goat anti-mouse IgG antibody (1:400, Abcam, ab97035) for 1 h. The nuclei were then stained with 4′, 6-diamidino-2-phenylindole (DAPI; Biosharp, Beijing, China) for 20 min. The number of total zygotes and BrdU-positive zygotes were counted to assess the frequency of BrdU-positive zygotes (number of BrdU-positive zygotes/total number of zygotes scored). The time point at which the frequency of BrdU-positive ≥ 10% was accepted as the starting time of S phase, and the time at which 90% BrdU-positive disappeared was accepted as the end of S phase. The time point when 95% of the zygotic embryos were cleaved represented the endpoint of M phase.

### Immunofluorescence Staining

Immunofluorescence staining was performed as previously described (Qian et al., 2016; Zhang et al., 2016). Zygotes were digested with Tyrode’s acid solution (Sigma, T1788) for 30 s to remove the zonae pellucidae, before being fixed in 4% paraformaldehyde for 30 min, and mounted on a polylysine-coated slide. Then, the zygotes were permeabilized with 0.5% Triton X-100 for 20 min, and blocked with PBS containing 3% BSA and 10% goat or donkey serum for 1 h. After that, samples were immunolabeled with primary antibody at 4°C overnight, followed by Alexa 488 (goat anti-rabbit, 1:400, Abcam, ab150077) or Cy 3 (goat anti-rabbit, 1:400, Abcam, ab6939) IgG secondary antibody for 1 h; the nuclei were stained with DAPI (Biosharp, Beijing, China) for 20 min. Quantification of fluorescence signal was performed with ImageJ software. Primary antibodies: rabbit anti-phosphor-AMPK (α, phospho Thr172; 1:200, Abcam, ab23875), rabbit anti-phospho-histone H2AX (phospho Ser139; 1:200, Abcam, ab2893), rabbit anti-phospho-histone H3 (phospho Ser10; 1:600, Cell Signaling Technology, #53348), and rabbit anti-p21 (1:100, Affinity, AF6290).

### AMPK Inhibition by Small Molecule Inhibitors

Both AMPK inhibitor Compound C (MCE) and SBI-0206965 (MCE), were used at a final concentration of 5 μM. At 5 hpi, zygotes were incubated with an embryo culture medium containing either 5 μM Compound C or 5 μM SBI-0206965. At 7 hpi, zygotes were treated with H_2_O_2_ (0.03 mM) for 30 min, followed by washing with fresh embryo culture medium. Embryos were then cultured in embryo culture medium containing either 5 μM Compound C or 5 μM SBI-0206965 at 37°C in a 5% CO_2_ incubator.

### Terminal Deoxynucleotidyl Transferase dUTP Nick End Labeling (TUNEL) Assay

The TUNEL assay was performed to assess blastocyst apoptosis, using the In Situ Cell Death Fluorescein Kit (Roche) in accordance with the manufacturer’s instruction. Blastocysts were collected at 108 hpi. The zonae pellucidae were first removed from the embryos with Tyrode’s acid solution (Sigma, T1788), embryos were then fixed in 4% paraformaldehyde for 30 min, and mounted on polylysine slides. Then, the embryos were permeabilized with 0.5% Triton X-100 for 30 min, and incubated with fluorescein-conjugated dUTP and terminal deoxynucleotidyl transferase at 37°C for 1 h in the dark. The reaction was terminated by washing with TPBS for 15 min, and then the nuclei of embryos were stained with DAPI. The apoptotic rate represents the percentage of TUNEL-positive cells relative to the total cell number of blastocysts (Qian et al., 2016).

### Western Blot

Four-hundred zygotes were used per lane for SDS-PAGE. Cells were lysed with SDS sample buffer (63 mM Tris-HCl, 10% glycerol, and 2% SDS) that contained protease and phosphatase inhibitors. The proteins of each sample were separated by SDS-PAGE and transferred onto PVDF membranes. Membranes were blocked with 5% non-fat milk in Tris-buffered saline-Tween 20 (TBST) for 1 h, and incubated with the specified primary antibodies overnight at 4°C. Then, membranes were incubated with the associated HRP-conjugated secondary antibodies (Jackson ImmunoResearch Laboratories) for 1 h, followed by extensive washing with TBST. After antibody incubation, an enhanced chemiluminescence substrate (Thermo) was added and the protein bands were visualized using a Bio-Rad ChemiDoc XRS+ Image station (Bio-Rad). The band intensity was quantified using ImageJ software. Primary antibodies: rabbit anti-phospho-AMPK (α, phospho Thr172; 1:1000, Abcam, ab23875), rabbit anti-AMPK (α, 1:1000, Cell Signaling Technology, #5832), mouse anti-phospho-histone H2AX (phospho S139; 1:1000, Abcam, ab26350), rabbit anti-H2AX (1:1000, Cell Signaling Technology, # 2595S), rabbit anti-p53 (1:500, Affinity, AF0879), rabbit anti-phospho-p53 (phospho Ser15; 1:500; Affinity, AF3075), rabbit anti-phospho-p53 (phospho Ser20; 1:500; Affinity, AF3073), rabbit anti-phospho-p53 (phospho Ser46; 1:500; Sigma-Aldrich, SAB4504503), rabbit anti-p21 (1:500, Affinity, AF6290), rabbit anti-CDK1 (1:1000, Abcam, ab18), rabbit anti-CDK1 (phospho Tyr15; 1:1000, Abcam, ab47594), rabbit anti-phospho-CDK1 (phospho Thr161; 1:1000, Abcam, ab201008), rabbit anti-CyclinB1 (1:1000, Abcam, ab227844), rabbit anti-Acetyl-CoA Carboxylase (1:1000, Cell Signaling Technology, #3662), rabbit anti-phospho-Acetyl-CoA Carboxylase (phospho Ser79; 1:1000, Signaling Technology, #11818), and mouse anti-β-actin (1:10,000, Sigma-Aldrich, A5441).

### Image Acquisition

Images of TUNEL-stained blastocysts were taken using a fluorescence microscope (Nikon Eclipse 90 Ni-E) with a 40 × objective. Image size was set to 1024 × 1024 pixels, gain, and digital gain to 0, exposure time to 100 ms. Images of other immunofluorescence staining were acquired on a confocal microscope (Zeiss 800) using a 40 × oil objective. Frame size was set to 1024 × 1024 pixels, pinhole to 1 AU. For the same antibody staining detection, the laser intensity, gain, digital gain, and digital offset settings were kept for all the acquisitions. Representative images of embryos at 24, 48, and 96 hpi were taken using an inverted microscope (Leica DMi8) with a 10 × objective.

### Quantification of the Fluorescence Signal

Image J software was used to measure the mean fluorescence intensity. To define the region of the nucleus for intensity measurements, the DNA channels (DAPI) were used as a mask and the mean fluorescence intensity of the nucleus was measured in each zygote. For the mean fluorescence intensity of the cytoplasm, the entire zygote along the inner side of the cell membrane was selected using the freehand selections tool in Image J, and the integrated density and area of each zygote was measured. The mean fluorescence intensity of the cytoplasm was calculated with the following formula: (integrated density of zygote – integrated density of nucleus)/ (area of zygote – area of the nucleus). The average mean fluorescence density of the background was measured in three different regions of the same area size of nucleus or cytoplasm for each image, and was subtracted in the measurement of mean fluorescence intensity of the nucleus and cytoplasm. All measurements were performed on the original, untreated acquisitions. All the representative images of immunofluorescence staining shown in the pictures were adjusted based on LUTs, which ensures consistent changes in the fluorescence intensity of the images in the same experiment.

### Statistical Analysis

Statistical analysis was performed using GraphPad Prism 8 software. Error bars indicate mean ± standard deviation (SD) unless otherwise specified. Data were assessed with the Student’s *t*-test (two groups) and one-way ANOVA followed by Tukey’s test (multiple groups). *p* < 0.05 was considered statistically significant. Significance was determined at ^∗^*p* < 0.05, ^∗∗^*p* < 0.01, and ^∗∗∗^*p* < 0.001.

## Results

### Fluorescent Staining of γH2AX in the Interphase of Mouse Zygotes Under Mild Oxidative Stress

**FIGURE 1:**
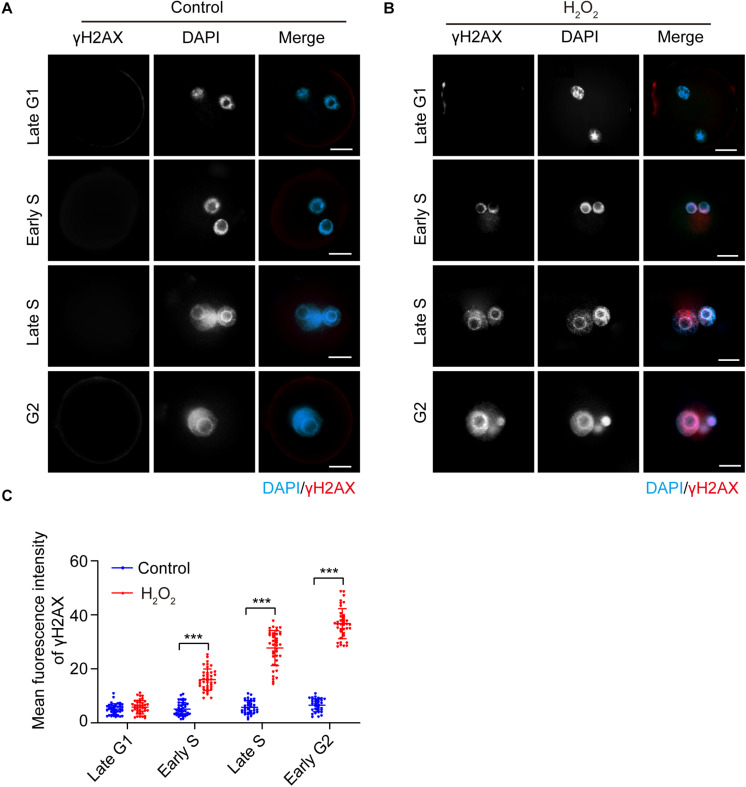
γH2AX immunostaining (red) detection in the interphase of mouse zygotes. Nuclei were stained with DAPI (blue). **(A)** Control group. Scale bar = 20 μm. **(B)** H_2_O_2_-treated group. Scale bar = 20 μm. **(C)** Quantification of the mean fluorescence intensity of γH2AX in both control and H_2_O_2_-treated zygotes at different stages during interphase. *N* = 40 zygotes per group at each stage. Results were analyzed by Student’s *t*-test. ***p < 0.001. Error bars: SD.

The presence of the DNA damage and repair marker γH2AX in the interphase of oxidatively stressed mouse zygotes was visualized using immunofluorescence staining. As shown in Figure 1, the γH2AX staining signal was undetectable in both H_2_O_2_-treated zygotes and control zygotes in the late G1 phase (8.5 hpi). In the early S phase (10.5 hpi), the γH2AX signal first occurred in the nuclei of the H_2_O_2_-treated zygotes and was undetectable in the control zygotes. The mean fluorescence intensity in the H_2_O_2_-treated zygotes was significantly higher than that of control zygotes. In the late S phase (17.0 hpi) and G2 phase (19.0 hpi), the γH2AX signal increased in H_2_O_2_-treated zygotes. However, the mean fluorescence intensity of zygotes in the control group was similar to those of control zygotes in the previous two stages, which was significantly lower than that of H_2_O_2_-treated zygotes.

### Fluorescent Staining and Subcellular Distribution of Activated AMPK in the Interphase of Mouse Zygotes Under Mild Oxidative Stress

**FIGURE 2:**
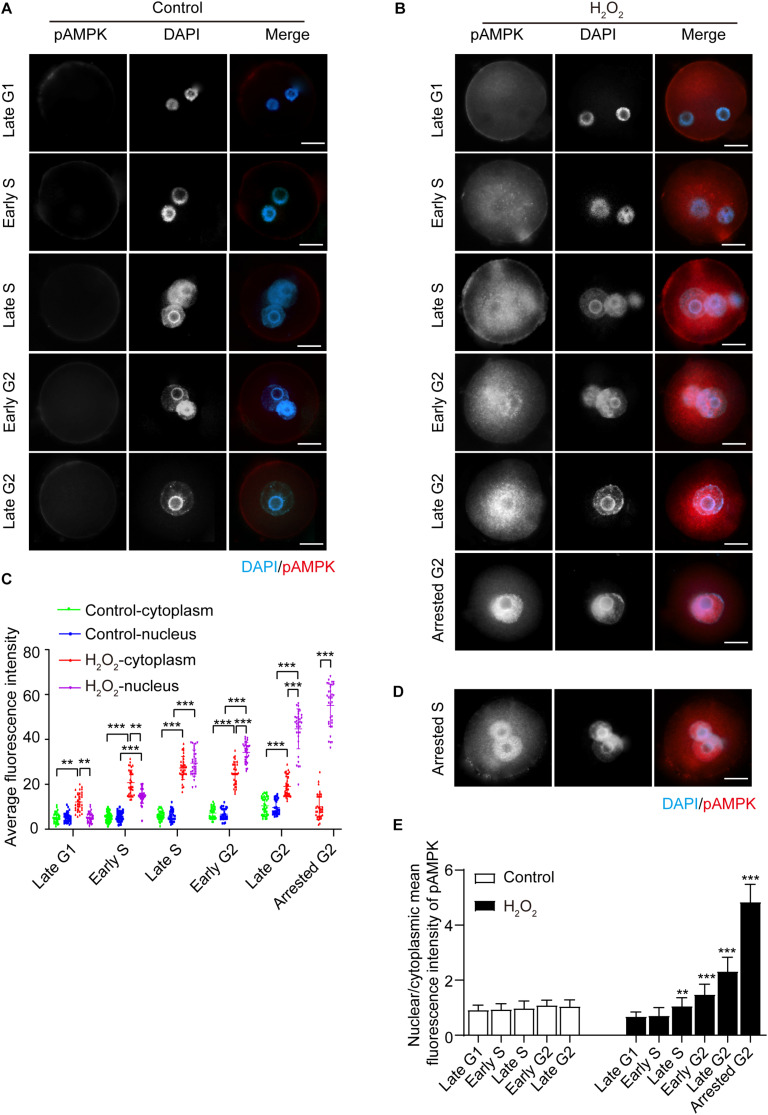
Subcellular distribution and presence of phosphorylated AMPK (pAMPK). pAMPK immunostaining (red) was visualized at different stages during mouse zygote interphase. Nuclei were stained with DAPI (blue). **(A)** Control zygotes. Scale bar = 20 μm. **(B)** H_2_O_2_-treated zygotes. Scale bar = 20 μm. **(C)** Mean fluorescence intensity of nuclear and cytoplasmic pAMPK in both control and H_2_O_2_-treated zygotes at different stages during interphase. *N* = 35 zygotes per group at each stage. **(D)** pAMPK immunostaining in H_2_O_2_-treated zygotes arrested in the S phase. Scale bar = 20 μm. **(E)** Changes in the nuclear/cytoplasmic ratio of the mean fluorescence intensity of pAMPK in control and H_2_O_2_-treated zygotes. *N* = 35 zygotes per group at each stage. **p < 0.01; ***p < 0.001 compared to late G1 phase within the group. Results in **(C,E)** were analyzed by one-way ANOVA and Tukey’s test. **p < 0.01; ***p < 0.001. Error bars: SD.

We then examined the effects of oxidative stress on AMPK phosphorylation of the α subunit at Thr172, which indicates AMPK activation (Novikova et al., 2015). We treated zygotes at 7 hpi with 0.03 mM H_2_O_2_ for 30 min, at which time they were in the G1 phase. We then detected the presence and subcellular localization of pAMPK during different phases of the interphase, and quantified the mean fluorescence intensity of nuclear and cytoplasmic pAMPK in control and H_2_O_2_-treated zygotes. In control zygotes, the pAMPK fluorescence signal was negligible, and the mean fluorescence intensity of pAMPK in the cytoplasm and nucleus appeared slightly increased from late G1 to late G2 phase (Figures 2A,C). In H_2_O_2_-treated zygotes, as shown in Figures 2B,C, the zygotes have a weak, punctuate pAMPK staining in the cytoplasm in the late G1 phase (8.5 hpi), while the mean fluorescence intensity of cytoplasmic pAMPK was significantly stronger than that of the control group. However, the nuclear pAMPK signal was no different from that of the control group and the mean cytoplasmic pAMPK intensity was significantly stronger than the nuclear pAMPK signal. In the early S phase (10.5 hpi), the pAMPK signal increased in the cytoplasm compared to H_2_O_2_-treated zygotes at the late G1 phase, and punctuate pAMPK staining was present in the nucleus. Moreover, the mean pAMPK fluorescence intensity in the cytoplasm and nucleus was significantly stronger than that of control zygotes and the cytoplasmic pAMPK was significantly stronger than nuclear pAMPK. In the late S phase (17.0 hpi), pAMPK signal continued to increase in both the cytoplasm and nucleus compared with the H_2_O_2_-treated zygotes in the previous two phases, and most cytoplasmic pAMPK was distributed around the nucleus. At this stage, both the mean fluorescence intensity of cytoplasmic and nuclear pAMPK was notably stronger than that of control zygotes and cytoplasmic pAMPK was similar to nuclear pAMPK. In early G2 phase (18.0 hpi) and late G2 phase (19.5 hpi), although both the mean fluorescence intensity of cytoplasmic and nuclear pAMPK were still significantly stronger than that of control zygotes, the nuclear pAMPK signal in H_2_O_2_-treated zygotes was increased and cytoplasmic pAMPK was decreased compared to H_2_O_2_-treated zygotes in late S phase. Moreover, the nucleus exhibited a more intense fluorescence than the cytoplasm in H_2_O_2_-treated zygotes. At 21.0 hpi, all control zygotes have ruptured their nuclear membranes and entered the M phase. However, 65.25 ± 4.79% H_2_O_2_-treated zygotes were arrested in the G2 phase. In these embryos we observed an intense pAMPK signal in the nucleus, but only faint staining in the cytoplasm. Indeed, the mean intensity of nuclear pAMPK was remarkably stronger than cytoplasmic pAMPK. In addition, we found that at 21.0 hpi, 4.50 ± 0.88% zygotes were arrested in the S stage. In these zygotes, most of the pAMPK signal accumulated in the nucleus (Figure 2D).

To better evaluate the changes in pAMPK staining signal in the cytoplasm and nucleus from the late G1 phase to the late G2 phase, we calculated the nuclear/cytoplasmic ratio of the mean fluorescence intensity of pAMPK in zygotes at each stage in both the control and H_2_O_2_-treated groups (Figure 2E). We still used the nuclear/cytoplasmic ratio of the mean fluorescence intensity here, but not the nuclear/cytoplasmic ratio of the integrated fluorescence intensity, because the nucleus is small in zygotes. Thus, the integrated intensity does not show very well the enhancement of the nuclear signal even if the change is obvious. In addition, the nuclei size is inconsistent from the G1 phase to the G2 phase. The results showed that the nuclear/cytoplasmic ratio of mean fluorescence intensity in H_2_O_2_-treated zygotes was significantly increased from the late S phase to the arrested G2 phase compared to the late G1 phase, while the early S phase was not changed compared to the late G1 phase, because the mean fluorescence intensity was increased both in the cytoplasm and nucleus (Figures 2C,E). There was no difference in the nuclear/cytoplasmic ratio of pAMPK in control zygotes in the interphase. These results suggested that the oxidative stress induced phosphorylation and thus activation of AMPK in mouse zygotes. AMPK was first activated in the cytoplasm and then translocated from the cytoplasm to the nucleus, where almost all the pAMPK was accumulated in G2-arrested zygotes.

### Oxidative Stress Induced G2 Arrest in Mouse Zygotes, Which Is Recovered Following Inhibition of the AMPK Activity

**FIGURE 3:**
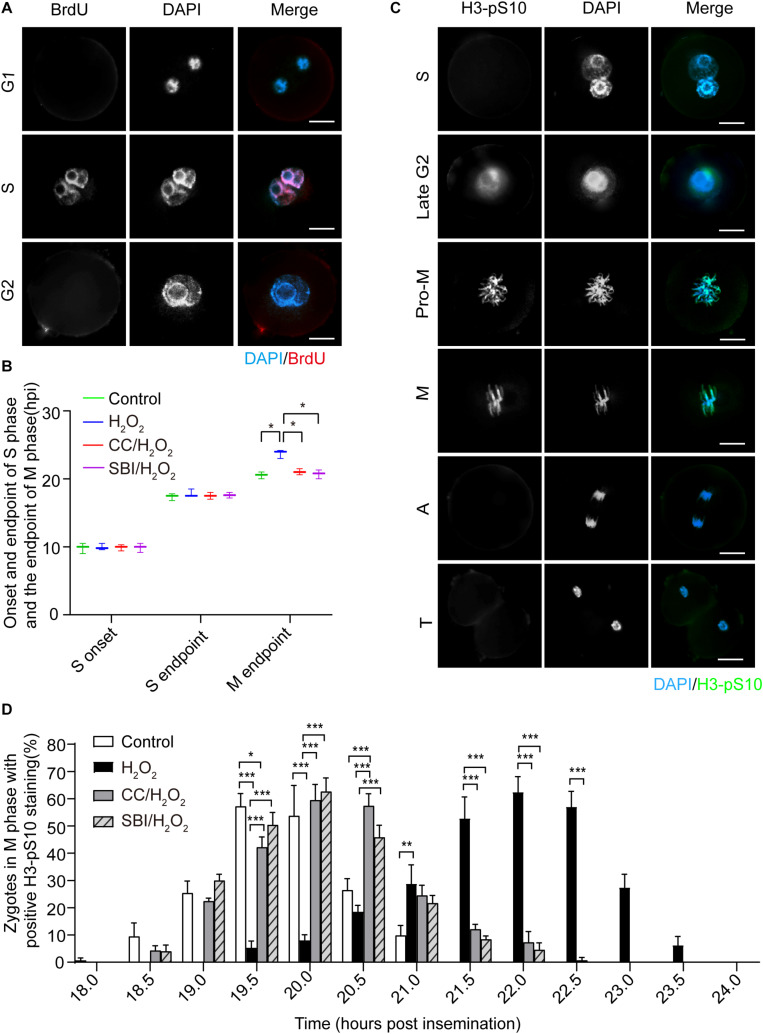
Oxidative stress induced a delay of mitosis entry in mouse zygotes. **(A)** BrdU signal (red) was positive in the S phase and negative in the G1 and G2 phases and nuclei were stained with DAPI (blue). Scale bar = 20 μm. **(B)** Determination of the onset and endpoint in the S phase and endpoint in the M phase of mouse zygotes. The onset and endpoint of the S phase were determined by BrdU incorporation. More than 60 zygotes per group were analyzed at each time point in each experiment (*n* = 3). The endpoint of the M phase was determined by detecting when zygotes divided. More than 150 zygotes were analyzed in each experiment (*n* = 3). CC, Compound C; SBI, SBI-0206965; hpi, hours post-insemination. **(C)** H3-pS10 signal (green). Nuclei were stained with DAPI (blue). Pro-M, M, A, and T refer to prometaphase, metaphase, anaphase, and telophase, respectively. Scale bar = 20 μm. **(D)** Percentage of zygotes with positive H3-pS10 staining in control, H_2_O_2_-treated, Compound C/H_2_O_2_-treated (CC/H_2_O_2_), and SBI-0206965/H_2_O_2_-treated (SBI/H_2_O_2_) zygotes from 18 to 24 hpi. More than 45 zygotes per group were immunostained and analyzed at each time point in each experiment (*n* = 3). Results in **(B,D)** were analyzed by one-way ANOVA and Tukey’s test. *p < 0.05; **p < 0.01; ***p < 0.001. Error bars: SD.

Next, we investigated the effect of oxidative stress on the cell cycle in mouse zygotes in the presence or absence of AMPK activity inhibition by Compound C and SBI-0206965. SBI-0206965 is a newly identified AMPK-specific inhibitor, which has a different set of potential off-target kinases, compared to Compound C (Dite et al., 2018). The onset and endpoint of the S phase were determined by BrdU incorporation experiment (Figure 3A). The endpoint of the M phase was determined by calculating the number of 2-cell embryos in each group of zygotes. We found that no significant difference in the onset and endpoint of the S phase in zygotes among control, H_2_O_2_-treated, Compound C/H_2_O_2_-treated, and SBI-0206965/H_2_O_2_-treated groups (Figure 3B). However, H_2_O_2_-treated zygotes exhibited a significant 3.1 h delay compared to control zygotes at the M phase endpoint (23.62 ± 0.51 hpi vs. 20.50 ± 0.42 hpi, *p* < 0.05), consistently with our previous study (Zhang et al., 2016). In Compound C/H_2_O_2_-treated and SBI-0206965/H_2_O_2_-treated zygotes, the M phase endpoint was 21.01 ± 0.36 and 20.75 ± 0.54 hpi respectively, both groups were significantly earlier than H_2_O_2_-treated zygotes (both *p* < 0.05), and slightly later than control zygotes, without statistical difference (both *p* > 0.05). These results indicated that mild oxidative stress caused a delay in the G2/M phase in the first cleavage of the zygotes, while the delay was abolished upon the inhibition of AMPK activity.

To further confirm the presence of a G2 block, we performed immunofluorescence staining of phosphorylated H3 histone (H3-pS10), a mitosis-specific marker. H3-pS10 is known to show intense and bright staining in the prometaphase/metaphase of the M phase, with a lower level of staining in heterochromatin in the late G2 phase (Hendzel et al., 1997), which was corroborated by our results (Figure 3C). H3-pS10 fluorescence staining was performed in zygotes of each group at 30 min intervals during the 18 ∼ 24 hpi interval. Then, we calculated the percentage of prometaphase/metaphase zygotes relative to the total zygotes, based on H3-pS10 staining, which was used as a marker of the entry in the M phase. We counted the percentage of H3-pS10-positive zygotes at each time point in control, H_2_O_2_-treated, Compound C/H_2_O_2_-treated, and SBI-0206965/H_2_O_2_-treated group (Figure 3D). We chose as critical time points when H3-pS10-positive zygotes begin to appear (beginning point) and when the percentage of H3-pS10-positive zygotes is > 50% (peak point), which indicate the beginning of the M phase and when most zygotes have entered the M phase, respectively. Results showed that the beginning point was 18.0 hpi in the control group and the peak points were at 19.5 and 20.0 hpi (57.24 ± 4.66 and 53.74 ± 11.16% respectively). At these two time points, there were 5.45 ± 2.31 and 8.13 ± 1.92% of H_2_O_2_-treated zygotes, respectively, significantly lower than those of the control group (both *p* < 0.001). Moreover, the percentage of H3-pS10-positive zygotes belonging to the control group at 19.5 hpi was higher than those belonging to the Compound C/H_2_O_2_-treated group (42.21 ± 3.77%, *p* < 0.05), while no significant difference was observed at 20.0 hpi compared to Compound C/H_2_O_2_-treated group. However, there was no statistical difference between the control group and the SBI-0206965/H_2_O_2_-treated group at these two time points. In the H_2_O_2_-treated group, the beginning point was 19.5 hpi, 1.5 h later than that of the control group, while the peak points were at 21.5, 22.0, and 22.5 hpi (52.83 ± 7.86, 62.45 ± 5.66, and 57.09 ± 5.65%, respectively), 2.0∼2.5 h later than that of the control group. At these peak points, H3-pS10-positive zygotes disappeared in the control group, whereas the percentage of H3-pS10-positive zygotes in Compound C/H_2_O_2_-treated zygotes (12.13 ± 1.81, 7.29 ± 3.97, and 0.73 ± 1.02%, respectively) and SBI-0206965/H_2_O_2_-treated zygotes (8.41 ± 1.22% at 21.5 hpi, 4.56 ± 2.53% at 22.0 hpi, and disappeared 22.5 hpi) significantly lower than those of the H_2_O_2_-treated group (*p* < 0.001 for all points).

Regarding zygotes in the Compound C/H_2_O_2_-treated group, the beginning point was 18.5 hpi, 1.0 h earlier than that of the H_2_O_2_-treated group, and 0.5 h later than that of the control group. The peak points were at 20.0 and 20.5 hpi (59.52 ± 5.73 and 57.39 ± 4.47%, respectively), 1.5∼2.0 h earlier than that of the H_2_O_2_-treated group and 0.5 h later than that of the control group. The percentage of H3-pS10-positive zygotes at 20.0 and 20.5 hpi was significantly higher than that of the H_2_O_2_-treated group (8.13 ± 1.92 and 18.66 ± 2.22% respectively, both *p* < 0.001), and at 20.5 hpi was significantly higher than that of the control group (26.47 ± 4.17%, *p* < 0.001). For SBI-0206965/H_2_O_2_-treated zygotes, the beginning point was also at 18.5 hpi, 1.0 h earlier than that of the H_2_O_2_-treated group, and 0.5 h later than that of the control group. The peak points were the same as that of the control group at 19.5 and 20.0 hpi (50.36 ± 4.62 and 62.64 ± 4.89%, respectively), 2.0∼2.5 h earlier than that of the H_2_O_2_-treated group. The percentage of H3-pS10-positive zygotes at these two points was significantly higher than that of the H_2_O_2_-treated group (5.45 ± 2.31 and 8.13 ± 1.92% respectively, both *p* < 0.001). These results suggested that mild oxidative stress induced a delay of mitosis entry, compatible with a G2 arrest. However, this arrest could be almost completely reversed by AMPK activity inhibition.

### Activated AMPK Contributes to Oxidative Stress-Induced Inactivation of CDK1 in Mouse Zygotes

**FIGURE 4:**
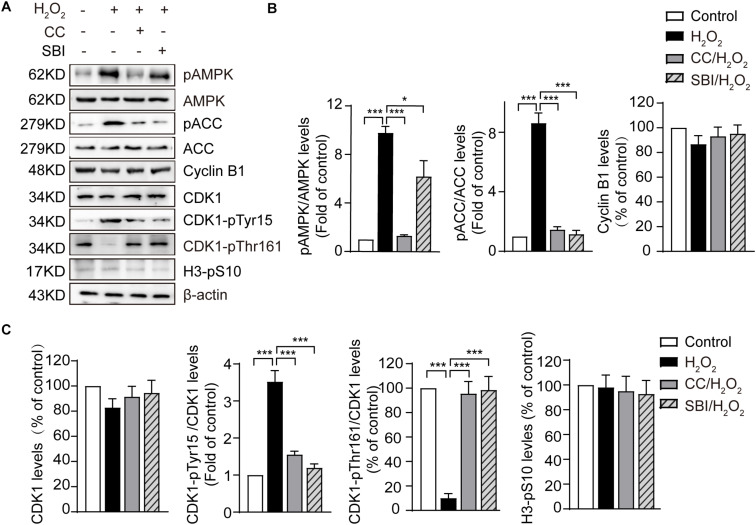
Activated AMPK contributes to oxidative stress-induced inactivation of CDK1 in mouse zygotes. CC, Compound C; SBI, SBI-0206965. **(A)** Representative data showing the level of pAMPK, pACC, Cyclin B1, CDK1, CDK1-pTyr15 and CDK1-pThr161 in control, H_2_O_2_-treated, Compound C/H_2_O_2_-treated, and SBI-0206965/H_2_O_2_-treated zygotes in the G2 phase. **(B,C)** Quantification data of figure (*n* = 3). Results were analyzed by one-way ANOVA and Tukey’s test. *p < 0.05; ***p < 0.001. Error bars: SD.

The most critical event in the transition from the G2 to the M phase is the activation of the Cyclin B1-CDK1 complex. This activation requires the dephosphorylation of CDK1 on its Thr14/Tyr15 residues and phosphorylation of the Thr161 conserved residue (Norbury et al., 1991; Morgan, 1995). We measured the levels of Cyclin B1, CDK1, CDK1-pTyr15, and CDK1-pThr161 by immunoblotting in the control, H_2_O_2_-treated, Compound C/H_2_O_2_-treated, and SBI-0206965/H_2_O_2_-treated zygotes in the G2 phase (the analysis was conducted at 19.0 hpi in control, Compound C/H_2_O_2_-treated, and SBI-0206965/H_2_O_2_-treated zygotes, and at 20.5–21.0 hpi in H_2_O_2_-treated zygotes). As shown in Figure 4, the protein levels of Cyclin B1 and CDK1 were slightly decreased in H_2_O_2_-treated zygotes, but the difference with control, Compound C/H_2_O_2_-treated, and SBI-0206965/H_2_O_2_-treated zygotes were not statistically significant. CDK1-pTyr15 levels were significantly increased in the H_2_O_2_-treated group, compared to those of the control group, but decreased upon AMPK activity inhibition with Compound C and SBI-0206965. Conversely, CDK1-pThr161 levels were dramatically decreased in the H_2_O_2_-treated group compared to those observed in the control group. However, this decrease was reversed upon the inhibition of AMPK activity. In addition, we measured the H3-pS10 levels in the three groups. The results showed weak levels among all the groups, with no statistically significant differences detected. This result further confirms that the analyzed zygotes were in the G2 phase, where a low level of H3-pS10 is expected.

In addition, we measured the levels of pAMPK and phosphorylation status of its substrate acetyl CoA carboxylase on Ser 79 site (pACC), which represents AMPK activation. The results showed that the levels of pAMPK were significantly increased in H_2_O_2_-treated zygotes compared to control zygotes, but decreased upon AMPK activity inhibition by Compound C and SBI-0206965, especially Compound C. Moreover, oxidative stress led to a significant increase in pACC, but this increase was fully abolished by treatment with Compound C and SBI-0206965. Conversely, we observed no significant difference in AMPK and ACC protein levels between the groups (Figures 4A,B), suggesting that the activation of AMPK contributed to oxidative stress-induced inactivation of CDK1, thereby affecting the activity of Cyclin B1-CDK1 complex.

### Activated AMPK Regulates Oxidative Stress Induction of Proteins Level of p53/p21 in Mouse Zygotes

**FIGURE 5:**
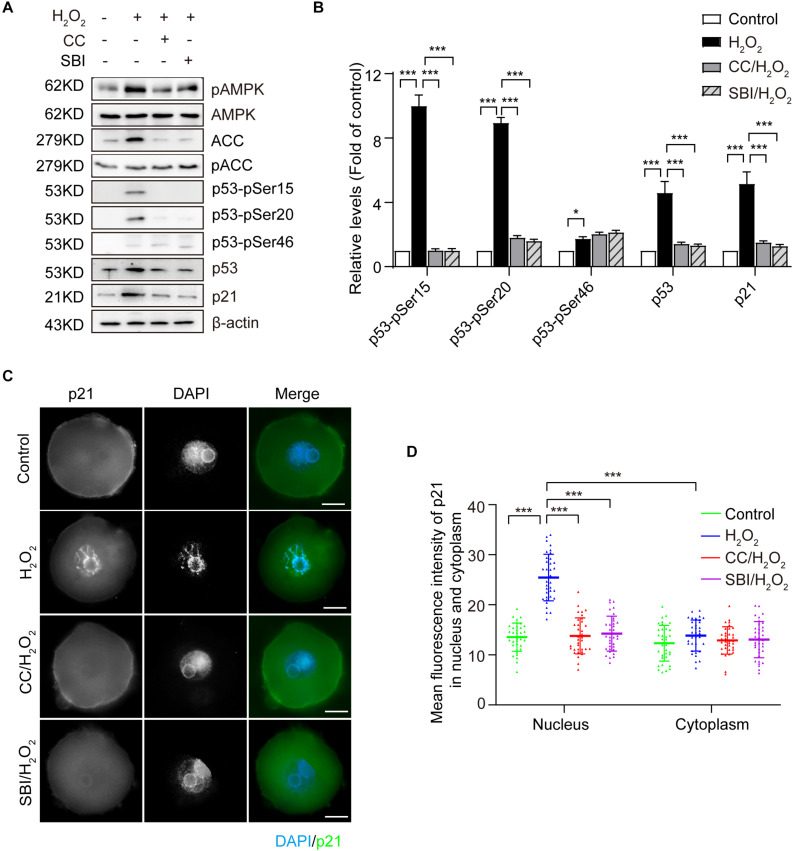
Activated AMPK regulates the oxidative stress induction of cell cycle regulatory proteins of p53 and p21 in mouse zygotes. CC, Compound C; SBI, SBI-0206965. **(A)** Representative data showing the level of p53, p21, p53-pSer15, p53-pSer20 and p53-pSer46 in control, H_2_O_2_-treated, Compound C/H_2_O_2_-treated, and SBI-0206965/H_2_O_2_-treated zygotes in the G2 phase. **(B)** Quantification data of figure (*n* = 3). **(C)** Representative image of the subcellular distribution and expression of p21 (green) using immunostaining. Nuclei were stained with DAPI (blue). Scale bar = 20 μm. **(D)** Mean fluorescence intensity of nuclear and cytoplasmic p21 in mouse zygotes in the G2 phase. *N* = 37 zygotes per group. Results in **(B**,**D)** were analyzed by one-way ANOVA and Tukey’s test. *p < 0.05; ***p < 0.001. Error bars: SD.

It has been reported that AMPK activation inhibits cell proliferation and induces cell cycle arrest in G2 by increasing the CKI protein p21 (Sanli et al., 2010; Xu et al., 2015; Qi et al., 2017). Moreover, the expression of p21 can be upregulated by p53-dependent and -independent mechanisms (Bunz et al., 1998; Sanli et al., 2010). To explore the AMPK-activating mechanisms contributing to the G2 arrest in mouse zygotes upon oxidative stress, we measured the protein levels of p53 and p21 in the G2 phase by immunoblotting. As shown in Figures 5A,B, oxidative stress resulted in a significant increase of p53 and p21, which was inhibited by blocking AMPK activity with Compound C and SBI-0206965. The stability and activity of p53 are tightly regulated by post-translational modifications, including phosphorylation. AMPK has been reported to phosphorylate p53 on Ser15 and Ser20, to stabilize p53 and increase its cellular levels (Jones et al., 2005; Maclaine and Hupp, 2009), and the AMPK-mediated p53 activation regulates the growth arrest at the G1/S and G2/M transitions, in response to genotoxic stresses. We measured the phosphorylation levels of p53 on Ser15 and Ser20 by immunoblotting. A non-AMPK-dependent phosphorylation site of p53, Ser46, which is phosphorylated by p38 and is associated with p53-dependent apoptosis (Oda et al., 2000), was used as a negative control. Our results (Figures 5A,B) showed that the treatment with H_2_O_2_ obviously increased the levels of p53-pSer15 and p53-pSer20, and these levels were significantly decreased upon the inhibition of AMPK activity with Compound C and SBI-0206965. In addition, oxidative stress caused a small amount of phosphorylation of p53-pSer46, which did not decrease after the inhibition of AMPK activity. These results suggested that, in oxidatively damaged mouse zygotes, the activation of AMPK regulates the cell cycle regulatory proteins p53 and p21. We observed an AMPK-dependent increase of p53 levels, which were associated with phosphorylation of p53 on Ser15 and Ser20.

We then used immunofluorescence staining to examine the subcellular localization of p21 in the G2 phase zygotes in each group. As shown in Figures 5C,D, we detected a weak uniform p21 signal in the cytoplasm and nucleus in the control zygotes. In the H_2_O_2_-treated group, the p21 signal in the nuclei increased dramatically and this increase was inhibited by blocking AMPK activity with Compound C and SBI-0206965. We calculated the mean immunofluorescence intensity of p21 in the cytoplasm and nucleus in each group. Results showed that the mean fluorescence intensity of nuclear p21 in the H_2_O_2_-treated group was significantly stronger than that of the control, Compound C/H_2_O_2_-treated and SBI-0206965/H_2_O_2_-treated group. However, we did not observe significant differences in the mean fluorescence intensity of cytoplasmic p21 among the four groups. Moreover, the mean fluorescence intensity of nuclear p21 was significantly stronger than the cytoplasmic intensity in H_2_O_2_-treated zygotes, while we observed no significant difference in the mean fluorescence intensity between nuclei and cytoplasm in the control, Compound C/H_2_O_2_-treated and SBI-0206965/H_2_O_2_-treated group.

### Inhibition of AMPK Activity Aggravated the Oxidative Stress-Induced DNA Damage of Zygotes and Apoptosis of Blastocysts

**FIGURE 6:**
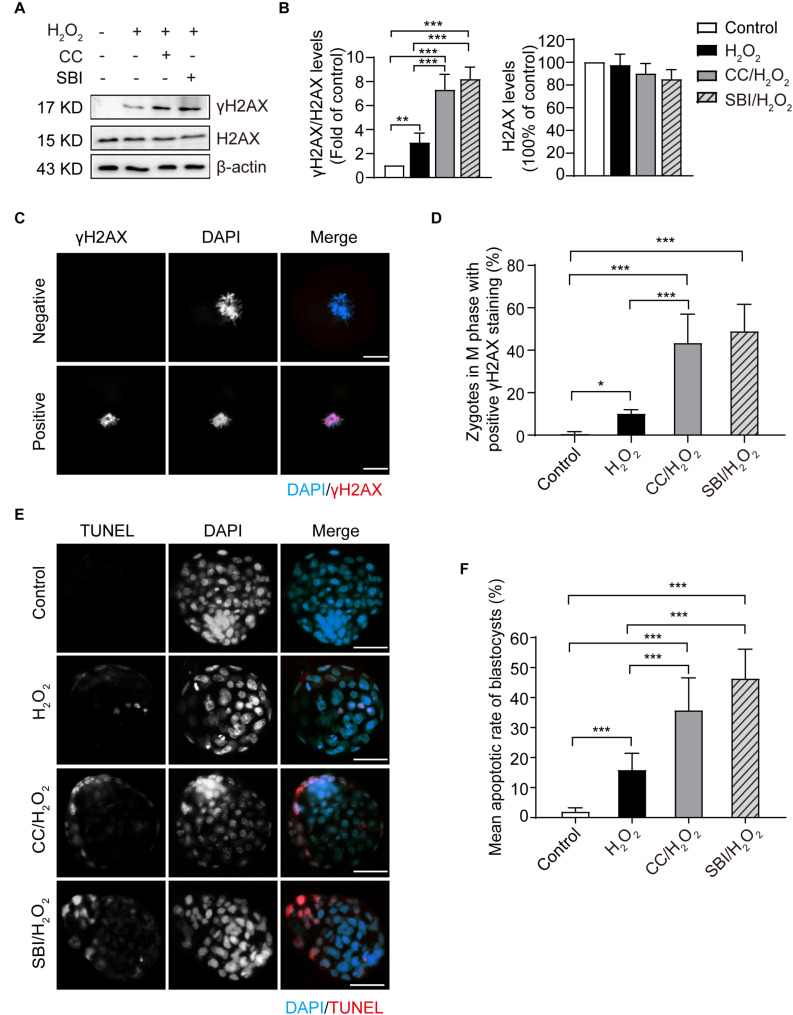
Inhibition of AMPK activity aggravated oxidative stress-induced DNA damage in zygotes and apoptosis in blastocysts. CC, Compound C; SBI, SBI-0206965. **(A)** Representative data of γH2AX levels in mouse zygotes in the M phase under oxidative stress. **(B)** Quantification of data from Figure 4A (*n* = 3). **(C)** γH2AX immunostaining (red) in zygotes in the M phase under oxidative stress. Nuclei were stained with DAPI (blue). Scale bar = 20 μm. **(D)** Quantification of zygotes at M phase with positive γH2AX immunostaining. More than 60 zygotes in each group were counted in each experiment (*n* = 6). **(E)** Detection of blastocyst apoptosis (red). Nuclei were stained with DAPI (blue). Scale bar = 100 μm. **(F)** Quantification of blastocyst apoptotic rate in the control group (*n* = 36), H_2_O_2_-treated group (*n* = 33) and Compound C/H_2_O_2_-treated group (*n* = 29), and SBI-0206965/H_2_O_2_-treated group (*n* = 19). Results in **(B,D,F)** were analyzed by one-way ANOVA and Tukey’s test. *p < 0.05; **p < 0.01; ***p < 0.001. Error bars: SD.

To investigate the effect of inhibiting AMPK activity on DNA damage, we examined the phosphorylation levels and immunofluorescence staining of γH2AX in the M phase of control, H_2_O_2_-treated, Compound C/H_2_O_2_-treated, and SBI-0206965/H_2_O_2_-treated zygotes (analyzed at 19.5, 22.0, 20.0, and 20.0 hpi respectively). As shown in Figures 6A,B, mild oxidative stress resulted in a significant increase in γH2AX levels (*p* < 0.01 compared to control group), and inhibition of AMPK activity with Compound C and SBI-0206965 significantly increased the accumulation of γH2AX (*p* < 0.001 compared to H_2_O_2_-treated group; *p* < 0.001 compared to control group for each group). This result was further confirmed by γH2AX fluorescence staining. As shown in Figures 6C,D, we found little or no γH2AX staining in the control zygotes (γH2AX foci-positive cells: 0.66 ± 0.94%). However, the proportion of γH2AX foci-positive cells increased significantly in H_2_O_2_-treated zygotes (10.20 ± 1.79%; *p* < 0.05 compared to control group), particularly in Compound C/H_2_O_2_-treated zygotes (43.29 ± 12.29%; both *p* < 0.001 compared to the control and H_2_O_2_-treated group) and SBI-0206965/H_2_O_2_-treated zygotes (49.78 ± 10.13%; both *p* < 0.001 compared to the control and H_2_O_2_-treated group). These results suggested that the inhibition of AMPK activity aggravated the oxidative stress-induced DNA damage in mouse zygotes.

In addition, we evaluated the blastocyst apoptosis rate in each group. As shown in Figures 6E,F, zygotes treated with 0.03 mM H_2_O_2_ had a significantly increased rate of apoptosis compared to the control group (15.85 ± 5.60 vs. 1.96 ± 1.30%, *p* < 0.001). Moreover, after inhibiting AMPK activity, the mean blastocyst apoptosis rate increased notably (35.76 ± 10.81% in the Compound C/H_2_O_2_-treated group, both *p* < 0.001 compared to the control and H_2_O_2_-treated group; 46.37 ± 9.76% in SBI-0206965/H_2_O_2_-treated group, both *p* < 0.001 compared to the control and H_2_O_2_-treated group). These results indicated that inhibition of AMPK activity increased the oxidative stress-induced apoptosis in blastocysts.

### Inhibition of AMPK Activity Decreased the Formation Rate of 4-Cell Embryos and Blastocysts Under Mild Oxidative Stress

**FIGURE 7:**
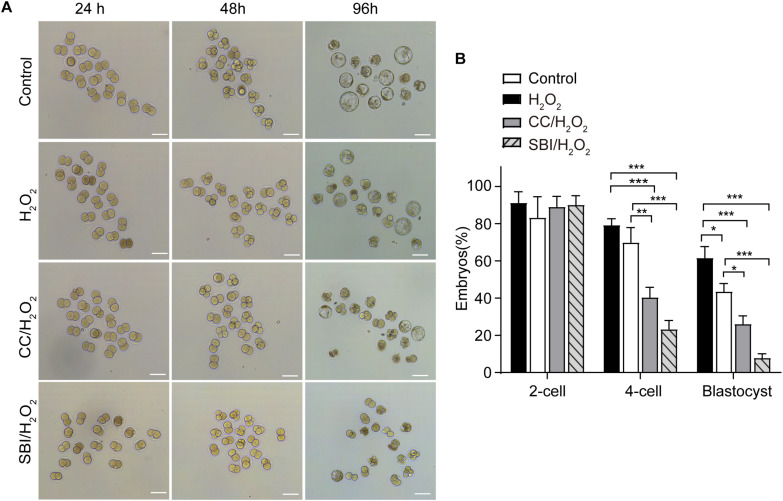
Inhibition of AMPK activity affected the development of mouse embryos under mild oxidative stress. CC, Compound C; SBI, SBI-0206965. **(A)** Representative image of embryos at 24, 48, and 96 hpi, coinciding with the formation of 2-cell embryos, 4-cell embryos, and blastocysts. Scale bars = 100 μm. **(B)** Statistics of 2- cell, 4- cell and blastocyst formation rate. More than 150 embryos in each experiment were counted (*n* = 3). Results were analyzed by one-way ANOVA and Tukey’s test. *p < 0.05; **p < 0.01; ***p < 0.001. Error bars: SD.

We evaluated the formation rates of 2-, 4-cell embryos, and blastocysts in the three groups analyzed at 24, 48, and 96 hpi, respectively (Figure 7A). Compared to the control and H_2_O_2_-treated zygotes, inhibition of AMPK activity with Compound C and SBI-0206965 significantly reduced 4-cell embryo and blastocyst formation rates, but did not affect the 2-cell embryo formation rate (Figures 7A,B). The 4-cell embryo formation rate in the Compound C/H_2_O_2_-treated group was 33.76% lower than the control group (*p* < 0.001), and 25.41% lower than the H_2_O_2_-treated group (*p* < 0.01). In the SBI-0206965/H_2_O_2_-treated group, the 4-cell embryo formation rate was 51.90% lower than the control group (*p* < 0.001), and 42.52% lower than the H_2_O_2_-treated group (*p* < 0.001).

Regarding the blastocyst formation rate, inhibition of AMPK activity with Compound C led to a 36.12% reduction compared to the control group (*p* < 0.001) and a 17.92% reduction compared to the H_2_O_2_-treated group (*p* < 0.05), inhibition of AMPK activity with SBI-0206965 led to a 50.72% reduction compared to the control group (*p* < 0.001) and a 32.66% reduction compared to the H_2_O_2_-treated group (*p* < 0.001). In addition, consistently with our previous findings (Qian et al., 2016; Wu et al., 2017), the blastocyst formation rate in the H_2_O_2_-treated group was 18.06% lower than that of the control group (*p* < 0.05), while we observed no statistical differences in the 2- and 4-cell embryo formation rate between the control and H_2_O_2_-treated groups (Figures 7A,B).

## Discussion

Reactive oxygen species generation in suboptimal culture conditions causes DNA damage in embryos cultured *in vitro*, which is an important cause of embryonic development arrest in IVF-derived embryos. Due to the wide application of human-assisted reproductive technology, it is of great importance to study DNA damage-dependent pathways in early embryos.

The development of the zygotes is controlled by maternal mRNA and protein that deposited in the oocytes (Tadros and Lipshitz, 2009; Langley et al., 2014). However, the function of G2/M checkpoints and DNA repair mechanisms in oocytes and zygotes may be different from somatic cells (Shimura et al., 2002; Yukawa et al., 2007; Homer et al., 2020). In the present study, we demonstrated that mild oxidative stress induces G2 arrest in mouse zygotes and that the DNA repair marker γH2AX is present since the early S phase. These results confirmed the presence of a G2/M checkpoint mechanism in mouse zygotes during the first cleavage, and that the G2/M checkpoint and DNA repair mechanisms seem to cope with mild oxidative stress. Our results differ from those published by Shimura et al. (2002) and Yukawa et al. (2007). Shimura et al. (2002) claimed that the G2/M checkpoint was absent in mouse zygotes, while Yukawa et al. (2007) showed that, although the G2/M checkpoint and DNA repair mechanisms are limited in mouse zygotes, treatment with γ rays could induce G2 arrest. Moreover, they found that γH2AX was presented in 4-cell embryos, morulae, and blastocysts, but not in the 1- and 2-cell stages. These discrepancies may be due to variations in the source, time, and extent of the damage. Shimura et al. (2002) fertilized mouse oocytes with sperm irradiated with 6 Gy of X-rays, obtained from a male mouse whose testicular area was irradiated with a dose rate of 1 Gy/min. Yukawa et al. (2007) treated the zygotes with 10 Gy of γ ray at a rate of 9.3 Gy/min at 12 hpi, coinciding with the G2 phase. In our study, we treated the zygotes with 0.03 mM H_2_O_2_ for 30 min at 7 hpi, coinciding with the G1 phase. In addition, mouse zygotes fertilized with severe sperm DNA damage induced by divalent cations treatment are characterized by G2 arrest and an increase of γH2AX foci formation (Gawecka et al., 2013), suggesting that the G2/M checkpoint and DNA repair mechanisms employed in mouse zygotes may be damage-specific. Here, the detection of γH2AX since early S phase supports studies reporting that both non-homologous end joining (NHEJ) and homologous recombination (HR) repair pathways are active in zygotes and are capable of some DNA repair (Matsuda et al., 1989; Derijck et al., 2008).

AMPK participates in many cellular processes, including metabolic regulation (Herzig and Shaw, 2017), autophagy (Pei et al., 2018), mitochondrial function (Herzig and Shaw, 2017), cell growth (Shaw, 2009), and genomic stability (Sanli et al., 2010, 2014). These functions rely on the ability of AMPK to phosphorylate a variety of substrates, regulating the expression of a large number of genes. The access to and modification of these substrates require a proper localization of AMPK. Our study describes the dynamics of AMPK activation and the cytoplasmic-nuclear transport of activated AMPK (pAMPK) in the interphase of H_2_O_2_-treated zygotes. We found that pAMPK is significantly increased from the G1 to the G2 phase in H_2_O_2_-treated zygotes. Moreover, in these zygotes, AMPK is first activated in the cytoplasm in the late G1 phase and the majority of pAMPK translocated to the nucleus in arrested G2 zygotes. This finding is consistent with what was reported by Kodiha et al. (2007), who found that cells increased the levels, as well as the nuclear/cytoplasm ratio, of pAMPK after recovery from energy depletion induced by deoxyglucose-NaN_3_. Here, we demonstrated that the nuclear accumulation of pAMPK might be associated with cell cycle regulation. However, our findings are somewhat different from those reported by Sanli et al. (2010). In their study, AMPK mediated the G2/M transition also in ionizing radiation-treated human cancer cells, but pAMPK was first activated in the nucleus and a small amount of pAMPK was subsequently translocated from the nucleus to the cytoplasm. This discrepancy may be explained by the use of different stressors and cell types. In addition, we observed that, in zygotes arrested in the S phase, the majority of pAMPK are in the nuclei, indicating that, in mouse zygotes under oxidative stress conditions, AMPK is not only involved in G2 arrest, but also in S arrest.

In the present study, we found that the γH2AX levels in the M phase are significantly increased in Compound C/H_2_O_2_-treated and SBI-0206965/H_2_O_2_-treated embryos, compared to H_2_O_2_-treated embryos. These results suggest that the activation of AMPK affects DNA damage repair by controlling the G2 cell cycle progression in mouse zygotes. It was reported that AMPK is involved in the NHEJ DNA repair pathways (Ui et al., 2014). However, whether AMPK participates in NHEJ in mouse zygotes under mild oxidative stress remains to be determined in future studies. In addition, abolishing G2 arrest by inhibiting AMPK activity significantly aggravates DNA damage in the M phase, which further demonstrates that the G2/M checkpoint and DNA damage repair mechanisms are functional in mouse zygotes under mild oxidative stress. Moreover, γH2AX levels are increased in H_2_O_2_-treated zygotes in the M phase compared to control zygotes. This may be due to the presence of continuous oxidative damage. Indeed, in our previous study we showed that there is a high level of ROS in the G2/M phase of H_2_O_2_-treated embryos (Huang et al., 2019).

The early stage of embryonic development, including fertilization and the very first cell division is controlled by maternal mRNAs and proteins that are deposited in the egg during oogenesis (Tadros and Lipshitz, 2009; Langley et al., 2014). Zygotic transcription [also known as zygotic gene activation (ZGA)] commences at the 2-cell stage in mouse zygotes, and is a vital process in the maternal to zygotic transition (Duncan and Schultz, 2010). Embryos develop the ability to support the proper development in the early stage during the ZGA phase (Langley et al., 2014). For the above reasons, the treatment with 0.03 mM H_2_O_2_ has more effect on the blastocysts rather than the 2- and 4-cell embryos, as observed in both our present and previous studies. The treatment with 0.03 mM H_2_O_2_ decreases the blastocysts formation rate, but the 2- and 4- cell formation rates are not affected (Qian et al., 2016). We observed that AMPK activation facilitates the development and survival of oxidative stress-damaged embryos. Conversely, inhibition of AMPK activity decreases the formation rate of 4-cell embryos and blastocysts and increased the apoptosis rate in blastocysts. This may partly be due to the increased DNA damage observed upon the inhibiting of AMPK activity. An incomplete repair of DNA damage in 1-cell embryos may lead to more severe DNA damage during later stages of embryonic development and can negatively affect the development and survival of embryos. In addition, activated AMPK affects the cellular function through a variety of processes aimed at restoring the energy balance, including metabolism, mitochondrial biogenesis, autophagy, and potency loss of stem cell and embryo (Steinberg and Kemp, 2009; Novikova et al., 2015; Bolnick et al., 2016, 2017; Herzig and Shaw, 2017; Pei et al., 2018). Moreover, the AMPK inhibitor we used, SBI-0206965, is also an inhibitor of the autophagic kinase ULK1 (Dite et al., 2018), which would inhibit autophagy of embryonic cells. These may also explain the decreased formation rate of 4-cell embryos and blastocysts and the increased apoptosis rate in blastocysts after inhibiting AMPK activity.

p21 can be regulated by growth-suppressive signals, such as p53 (Bunz et al., 1998). Nevertheless, it was reported that p21 could also be upregulated through an AMPK-dependent pathway in p53-null cells (Sanli et al., 2010). We observed that the protein levels of p53 and p21 are significantly induced by oxidative stress in mouse zygotes in the G2 phase, and this effect is abolished in the presence of the AMPK antagonist Compound C and SBI-0206965. These results suggest that AMPK regulates the oxidative stress induction of p53 and p21, and that p21 is upregulated in a p53-dependent manner. These results are consistent with previous studies (Zhou et al., 2009; Sanli et al., 2010). In unstressed cells, p53 interacts with Mdm2 and MdmX, which inactivate p53, and ubiquitinate it activating the proteasome-mediated degradation, keeping p53 at a low level (Appella and Anderson, 2001). In response to stresses, most known post-translational modifications of p53, including phosphorylation, are induced. These modifications disrupt the interaction between p53 and its negative regulators, leading to the activation and stabilization of p53 (Appella and Anderson, 2001; Liu and Xu, 2011). In this study, we found that oxidative stress induced a significant increase in the phosphorylation of p53 on Ser15 and Ser20, which was AMPK-dependent. These results are consistent with previous studies, suggesting that AMPK directly phosphorylates p53 on Ser15 and Ser20 to stabilize and increase p53 protein levels (Jones et al., 2005; Maclaine and Hupp, 2009).

The ability of p21 to regulate cell cycle is dependent on its nuclear localization: cytoplasmic retention of p21 resulted in the loss of cell cycle inhibition and the gain of apoptosis activity (El-Deiry, 2001; Wu et al., 2011). Our results showed that, in the nucleus of mouse zygotes under mild oxidative stress, p21 levels mainly increased, and that the increase is abolished upon AMPK activity inhibition. These results indicate that AMPK-mediated G2 arrest in mild oxidative damaged mouse zygotes causes an increase of nuclear p21.

CDK1 activity is of utmost importance in the G2/M transition. Its activation requires dephosphorylation of its Thr14/Tyr15 residues by Cdc25 C, and binding to Cyclin B1, and phosphorylation of the Thr161 residue by the Cdk-activating kinase CAK (Norbury et al., 1991; Morgan, 1995). We show here that CDK1-Tyr15 is increased, while CDK1-Thr161 is decreased significantly in H_2_O_2_-treated zygotes. These effects were reversed after inhibition of AMPK activity, indicating that oxidative stress induced the inactivation of CDK1, and this inactivation is AMPK-dependent. The inactivation of CDK1 in H_2_O_2_-treated zygotes may be due to the increased level of p21, which inhibits the activity of CDK1 by restricting its activation by Cdc25 and CAK phosphatases (Smits et al., 2000; Charrier-Savournin et al., 2004; Lin et al., 2011). It was reported that p53 regulates 14-3-3σ in ionizing radiation-induced DNA damage, which binds and prevents CDK1 dephosphorylation by Cdc25C (Hermeking et al., 1997). In addition, p53 can bind directly to the Cdc25C promoter, downregulating Cdc25C expression (St Clair et al., 2004). Therefore, increased p53 levels may also explain the observed increased phosphorylation of CDK1 on Tyr15 in H_2_O_2_-treated zygotes.

**FIGURE 8:**
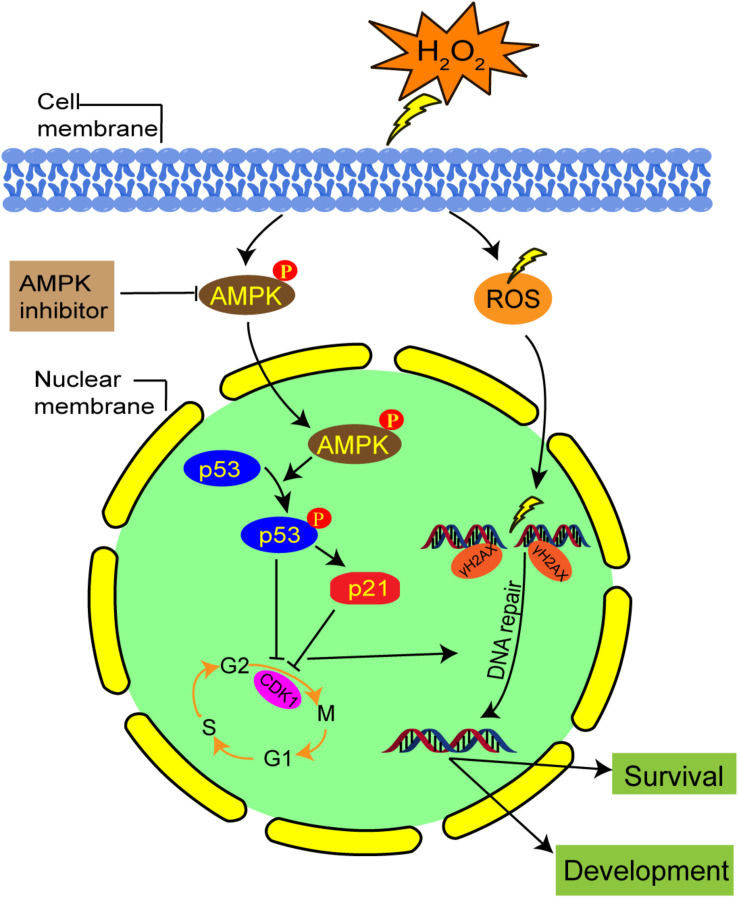
Schematic representation of the AMPK-mediated G2 arrest regulation and DNA damage in mouse zygotes under mild oxidative stress. Oxidative stress activated AMPK first in the cytoplasm, which is subsequently translocated in the nucleus. AMPK activation contributes to oxidative stress-induced G2 arrest, by inhibiting CDK1 activity via the upregulation protein level of p53 and p21. The G2 arrest facilitates DNA damage repair and the development and survival of oxidative stress-damaged embryos.

Our results suggest that the activation of AMPK contributes to G2 arrest via the p53/p21 pathway in mild oxidatively damaged mouse zygotes (Figure 8). However, these findings are different from what was reported by Macip et al. (2006), whose results showed that oxidative stress induced G2 arrest in p53-null tumor cells through a Chk1-dependent mechanism, and G1 arrest in p53 wild type cells in a p53-dependent way. This discrepancy may be explained by the use of different types of stress. Macip et al. (2006)’ research exposed cells to 100 μM tBH, an organic hydroperoxide, to induce oxidative stress. Instead, we generate oxidative stress by treating zygotes with 0.03 mM H_2_O_2_. Most importantly, different cell types will generate different damage responses to stress. Macip et al. (2006) used tumor cells, which have normal G1/S and G2/M checkpoints, while we used mouse zygotes, which have no traditional G1/S checkpoint.

In summary, in this study we showed that mild oxidative stress induced a G2 arrest in mouse zygotes, and activated AMPK contributed to this arrest. The underlying mechanism might be linked to CDK1 inhibition through the AMPK-p53/p21 pathway. Activation of AMPK may also regulate cell cycle progression, thereby affecting DNA repair, and facilitating the development and survival of oxidative stress-damaged embryos. Despite strong evidences of AMPK involvement, there is always the possibility that pharmaceutical inhibitors will affect non-specific targets other than AMPK. Further research in an AMPK null context is required to confirm the role of AMPK on G2 arrest and DNA repair in zygotes, which would definitely overcome the limits of such inhibitors.

## Data Availability Statement

All datasets generated for this study are included in the article.

## Ethics Statement

The animal study was reviewed and approved by Laboratory Animal Ethics Committee of our institution (SUMC2019-381).

## Author Contributions

PH and ZL conceived and designed the experiments and wrote the manuscript. PH and FX analyzed the data. All authors performed the experiments and read and approved the final manuscript.

## Conflict of Interest

The authors declare that the research was conducted in the absence of any commercial or financial relationships that could be construed as a potential conflict of interest.
